# Assessment of Ramucirumab plus paclitaxel as switch maintenance versus continuation of first-line chemotherapy in patients with advanced HER-2 negative gastric or gastroesophageal junction cancers: the ARMANI phase III trial

**DOI:** 10.1186/s12885-019-5498-3

**Published:** 2019-03-29

**Authors:** Maria Di Bartolomeo, Monica Niger, Federica Morano, Salvatore Corallo, Maria Antista, Stefano Tamberi, Sara Lonardi, Samantha Di Donato, Rossana Berardi, Mario Scartozzi, Giovanni Gerardo Cardellino, Francesco Di Costanzo, Lorenza Rimassa, Alberto Gianluigi Luporini, Raffaella Longarini, Alberto Zaniboni, Alessandro Bertolini, Gianluca Tomasello, Graziella Pinotti, Giorgio Scagliotti, Giampaolo Tortora, Andrea Bonetti, Andrea Spallanzani, Giovanni Luca Frassineti, Davide Tassinari, Francesco Giuliani, Saverio Cinieri, Evaristo Maiello, Claudio Verusio, Sergio Bracarda, Vincenzo Catalano, Michele Basso, Libero Ciuffreda, Ferdinando De Vita, Hector Soto Parra, Lorenzo Fornaro, Marta Caporale, Filippo de Braud, Filippo Pietrantonio

**Affiliations:** 1 Department of Medical Oncology, Fondazione IRCCS Istituto Nazionale Tumori, via G. Venezian, 1, 20133 Milan, Italy; 2 Department of Medical Oncology, Istituto Scientifico Romagnolo per lo Studio e la Cura dei Tumori (IRST), Ravenna Viale Randi, 5, 48121 Ravenna, Italy; 3 Department of Medical Oncology, IOV Istituto Oncologico Veneto, Via Gattamelata, 64, 35128 Padova, PD Italy; 4 Sandro Pitigliani Medical Oncology Department, Nuovo Ospedale di Prato, Via Suor Niccolina Infermiera, 20, 59100 Prato, Italy; 5 Department of Medical Oncology, AOU Ospedali Riuniti Di Ancona, via Corridoni, 11, 60123 Ancona, Italy; 6 Department of Medical Oncology, AOU Cagliari, Via Ospedale, 54, 09124 Cagliari, Italy; 7 Department of Medical Oncology, Azienda Sanitaria Universitaria Integrata di Udine, Via Pozzuolo, 330 – 33100, piazzale Santa Maria della misericordia 15, 33100 Udine, Udine Italy; 8 Department of Medical Oncology, AOU Careggi di Firenze, Largo Brambilla, 3, 50134 Florence, Italy; 9 Medical Oncology and Hematology Unit, Humanitas Cancer Center, Humanitas Clinical and Research Center, Via Alessandro Manzoni, 56, 20089 Rozzano, Milan Italy; 10 Department of Medical Oncology, IRCCS Policlinico San Donato, Piazza Edmondo Malan, 2, 20097 San Donato Milanese, MI Italy; 11 Department of Medical Oncology, Ospedale San Gerardo, Via G. B. Pergolesi, 33, 20900 Monza, Italy; 12 Department of Medical Oncology, Fondazione Poliambulanza, Via Leonida Bissolati, 57, 25124 Brescia, Italy; 13 Department of Medical Oncology, ASST della Valtellina e dell’Alto Lario, Via Stelvio, 25, 23100 Sondrio, Italy; 14 Department of Medical Oncology, Ospedale di Cremona, Viale Concordia, 1, 26100 Cremona, Italy; 15 Department of Medical Oncology, Ospedale di Circolo e Fondazione Macchi, Viale Luigi Borri, 57, 21100 Varese, Italy; 16 Department of Medical Oncology, AOU San Luigi Gonzaga, Regione Gonzole, 10, 10043 Orbassano, Torino Italy; 17 Department of Medical Oncology, AOUI Verona Ospedale Policlinico ‘Giambattista Rossi’ di Borgo Roma, Piazzale L.A. Scuro, 10, 37134 Verona, VR Italy; 18 Department of Medical Oncology, Ospedale Mater Salutis, Via Carlo Gianella, 1, 37045 Legnago, Verona, Italy; 19 Department of Medical Oncology, AOU di Modena, Via Emilia Est, 583-585, 41122 Modena, MO Italy; 20 Department of Medical Oncology, Istituto Scientifico Romagnolo per lo Studio e la Cura dei Tumori (IRST) IRCCS, via P. Maroncelli, 40, 47014 Meldola, Italy; 21 Department of Medical Oncology, Ospedale degli infermi di Rimini, Viale L. Settembrini, 2, 47923 Rimini, Italy; 22 Department of Medical Oncology, I.R.C.C.S. Istituto Tumori Bari, Viale Orazio Flacco, 65, 70124 Bari, Italy; 23 Department of Medical Oncology, Ospedale A. Perrino di Brindisi, Strada Statale 7 per Mesagne, 72100 Brindisi, Italy; 24 Department of Medical Oncology, Casa Sollievo della Sofferenza, Viale Cappuccini, 1, 71013 San Giovanni Rotondo, FG Italy; 25 Department of Medical Oncology, ASST Valle Olona, PO Saronno Piazzale Borella 1, 21047 Saronno, Varese Italy; 26 Department of Medical Oncology, Ospedale San Donato, Azienda USL Toscana Sudest Via Pietro Nenni, 20/22, 52100 Arezzo, Italy; 27 Department of Medical Oncology, Azienda Ospedaliera “Ospedali Riuniti Marche Nord”, Piazzale Cinelli, 4, 61121 Pesaro, Italy; 28 Department of Medical Oncology, Fondazione Policlinico Universitario “A. Gemelli” - IRCCS, Università Cattolica del Sacro Cuore, Largo Agostino Gemelli, 8, 00168 Rome, Italy; 29 Department of Medical Oncology, A.O.U. Citta della Salute e della Scienza di Torino, H Molinette, corso Bramante, 88, 10126 Torino, Italy; 30 Division of Medical Oncology, Department of Precision Medicine, University of Campania ‘Luigi Vanvitelli’ - School of Medicine, Via S.Pansini, 5, 80131 Naples, Italy; 31 Department of Medical Oncology, P.O. G. Rodolico, Via Plebiscito, 628 Catania, Italy; 32 Department of Medical Oncology, AOU Pisana, Polo Oncologico - Osp. S. Chiara, via Roma 67, 56100 Pisa, Italy; 33 Department of Hematology-Oncology, University Milan, Milan, Italy

**Keywords:** Metastatic gastric cancer, First line, Maintenance, Ramucirumab, Clinical trial

## Abstract

**Background:**

Platinum/fluoropyrimidine regimens are the backbone of first-line chemotherapy for advanced gastric cancer (AGC). However response rates to first line chemotherapy range from 30 to 50% and disease progression occurs after 4–6 cycles. The optimal duration of first-line therapy is still unknown and its continuation until disease progression represents the standard. However this strategy is often associated with cumulative toxicity and rapid development of drug resistance. Moreover, only about 40% of AGC pts. are eligible for second-line treatment.

**Methods:**

This is a randomized, open-label, multicenter phase III trial. It aims at assessing whether switch maintenance to ramucirumab plus paclitaxel will extend the progression-free survival (PFS) of subjects with HER-2 negative AGC who have not progressed after 3 months of a first-line with a platinum/fluoropyrimidine regimen (either FOLFOX4, mFOLFOX6 or XELOX). The primary endpoint is to compare Progression-Free Survival (PFS) of patients in ARM A (switch maintenance to ramucirumab and placlitaxel) versus ARM B (continuation of the same first-line therapy with oxaliplatin/fluoropyrimidine). Secondary endpoints are: overall survival, time-to-treatment failure, overall response rate, duration of response, percentage of patients that will receive a second line therapy according to arm treatment, safety, quality of life. Exploratory studies including Next-Generation Sequencing (NGS) in archival tumor tissues are planned in order to identify potential biomarkers of primary resistance and prognosis.

**Discussion:**

The ARMANI study estimates if patients treated with early swich with ramucirumab plus paclitaxel received benefit when compared to those treated with continuation of first line therapy. The hypothesis is that the early administration of an active, non-cross resistant second-line regimen such as ramucirumab plus paclitaxel may prolong the time in which patients are progression-free, and consequently have a better quality of life. Moreover, this strategy may rescue all those subjects that become ineligible for second-line therapy due to the rapid deterioration of health status after the first disease progression.

**Trial registration:**

ARMANI is registered at ClinicalTrials.gov (NCT02934464, October 17, 2016) and EudraCT(2016–001783-12, April 202,016).

## Background

Gastric cancer (GC) is the 4th most common cancer and the second leading cause of cancer-related death with 700,000 deaths reported annually, with higher incidence rates in Asia, Costa Rica, Peru, and Eastern Europe [1]. The five-year survival rate of gastroesophageal adenocarcinoma is < 30% for all stages and < 4% for metastatic disease [2, 3]. Systemic chemotherapy is the standard treatment for HER2 negative, advanced gastric or gastroesophageal junction (GEJ) cancers; first-line chemotherapy generally consists of a fluroropyrimidine and a platinum (cisplatin or oxaliplatin) combination regimen [4].

The V325 Phase 3 trial showed that the addition of docetaxel to cisplatin and 5FU combination improved progression free survival (PFS), overall response rate (ORR) with a small improvement in median survival (median 9.2 months and 8.6 months, respectively, *p* = 0.02), with increased toxicity [5]. Cunningham et al. in a large, multicenter, randomized Phase 3 study evaluated 4 regimens in first line: epirubicin + oxaliplatin + 5-fluorouracil (5-FU) [EOF], epirubicin + cisplatin + capecitabine [ECX], epirubicin + cisplatin + 5-FU [ECF], epirubicin + oxaliplatin + capecitabine [EOX]. No significant differences were observed in terms of response rate or PFS. Overall survival time was significantly longer among patients receiving EOX versus ECF (9.9 months, 9.3 months, 9.9 months, and 11.2 months for ECF, EOF, ECX, and EOX, respectively) [6]. Capecitabine was shown to be non-inferior to fluorouracil in terms of progression-free survival and overall survival [7]. A randomized phase 3 study compared epirubicin, cisplatin and capecitabine (ECX) with5-FU, l-leucovorin and irinotecan (FOLFIRI) as first line treatment in patients with advanced gastric or GEJ cancers. Patients were treated until disease progression or unacceptable toxicity. The results demonstrated no differences in response rate, median progression-free survival (PFS), and overall survival (OS) between the two treatments, except for time to treatment failure (TTF) that was significantly longer in FOLFIRI arm (median TTF: 4.24 vs. 5.1 months; *p* = 008). A second line therapy was administered only in 39% of patients treated with FOLFIRI versus 48% of patients treated with ECX. Approximately only 19% of patients received third line treatment [8].

In the past years two targeted therapies have been approved so far by the FDA in USA and EMA in EU for advanced GC (AGC): Trastuzumab and Ramucirumab. Trastuzumab is a monoclonal antibody targeting a tumor molecular alteration, that is HER2 overexpression/amplification, found in 10–15% of AGC. It has been shown to have survival benefit in combination with cisplatin/fluoropyrimidine for HER2 positive GC [9]. On the other hand, ramucirumab is a human IgG1 monoclonal antibody targeting Vascular Endothelial Growth Factor Receptor 2 (VEGFR2), and it is the first biological treatment given as a single drug that has survival benefits in patients with HER2 negative AGC or GEJ adenocarcinoma progressing after first-line chemotherapy. Ramucirumab was approved in many countries for the treatment of AGC refractory to treatment with fluoropyrimidines and platinum (with or without an anthracycline).based on the positive results of two randomized, double-blind, placebo-controlled, phase III studies. In the REGARD trial, patients progressing after first-line therapy with platinum/fluoropyrimidine regimens were treated with ramucirumab versus placebo [10], while in the RAINBOW trial they were given the combination of ramucirumab plus paclitaxel versus placebo plus paclitaxel [11]. These trials reported a median OS (mOS) of 5.2 months vs 3.8 months (hazard ratio [HR] 0.776, 95% CI 0.603–0.998; *p* = 0.047) and a mOS of 9.6 months vs 7.4 months (HR 0.807, 95% CI 0.678–0.962]; *p* = 0.017), respectively [12]. Of note, treatment with ramucirumab was associated with improved quality of life results and longer time to clinical deterioration in both the REGARD and the RAINBOW studies. As far as adverse events (AEs) are concerned, the REGARD trial reported similar AEs in the two treatment groups, with the exception of a higher incidence of hypertension in the ramucirumab arm. The combination of ramucirumab and paclitaxel, in the RAINBOW trial, resulted in a higher incidence of some ≥ G3 adverse events (AEs), such as neutropenia, hypertension and fatigue. Ramucirumab is now considered to be a standard second-line therapy of AGC in many countries.

Additionally, the results of the phase III RAINFALL study were recently published [13]. The objective of the study was to evaluate the benefit, in term of PFS, of ramucirumab in combination with fluoropyrimidine and cisplatin as first line therapy as compared to fluoropyrimidine and cisplatin alone in patients with AGC. The primary endpoint was met, as there was a very modest but statistically significant increase in median PFS for ramucirumab plus chemotherapy versus chemotherapy alone (5.7 vs. 5.4 months; HR 0.753, 95% CI [0.607, 0.935]; *p* = 0.0106). However, there was no difference in mOS (11.2 vs. 10.7 months; HR 0.962, 95% CI [0.801, 1.156]; *p* = 0.6757) nor in overall response rate (41.1% vs. 36.4%; *p* = 0.17) or disease control rate (81.9% vs. 76.5%; *p* = 0.095). Among randomized patients, only 50% of patients were treated in second line. However the post-progression treatment (ramucirumab as second line therapy) contributed to jeopardize the survival advantage. Based on these disappointing results, ramucirumab will not be pursued for a first-line indication in GC.

In summary, platinum/fluoropyrimidine regimens represent the backbone of first-line chemotherapy for AGC; the addition of a third chemotherapeutic agent was either associated with an increase in toxicity (i.e, docetaxel), or with a not-demonstrated superiority when compared to doublets [14]. Therefore, the use of triplet regimens in the first-line treatment of gastric cancer patients is limited and not standard. Additionally, clinical responses to first-line treatments range from 30% up to 50%, but disease progression occurs after a median of 4–6 cycles of chemotherapy. Only about 40% of the potential trial population is eligible for second-line treatment, whichever the first-line therapy is.

Continuation of fist-line chemotherapy until disease progression or unacceptable toxicity is consistent with observations in clinical practice, published international guidelines and phase III clinical trials in AGC [5, 7, 15, 16] .There is currently no approved treatment as maintence therapy following first-line treatment for gastric cancer. Since standard of care is not yet established in this setting, best supportive care (BSC) or continuation of fluoropyrimidine are accepted after a lead-in chemotherapy with platinum /fluoropyrimidine association.

Recently, switch maintenance for initial treatment of AGC with immunotherapy has already been conducted in the JAVELIN Gastric 100 study, which results are not mature yet [17].

The ARMANI study is designed to define whether switch maintenance with ramucirumab plus paclitaxel will extend PFS of subjects affected by with HER-2 negative AGC who have not progressed after a first-line platinum/fluoropyrimidine regimen, as compared to continuation of first-line chemotherapy.

## Methods

### Aims

Primary objective of this study is to compare PFS of subjects who receive switch maintenance therapy with ramucirumab plus paclitaxel (arm A) following a first-line chemotherapy doublet combination versus subjects who receive continuation of first-line chemotherapy until progressive disease,unacceptable toxicity, patient’s withdrawal consent or death (arm B).

Secondary objectives of this trial are to evaluate OS, time-to-treatment failure (TTF), overall response rate (ORR) and duration of response (DOR) of patients who receive switch maintenance (arm A) versus patients who receive continuation therapy (arm B). Additionally, the study will compare the percentage of patients that will receive a second line therapy according to arm treatment and it will evaluate safety (according to CTCAE v 4.03) and quality of life (patients reported outcomes [PRO]).

### Trial design

**Fig. 1:**
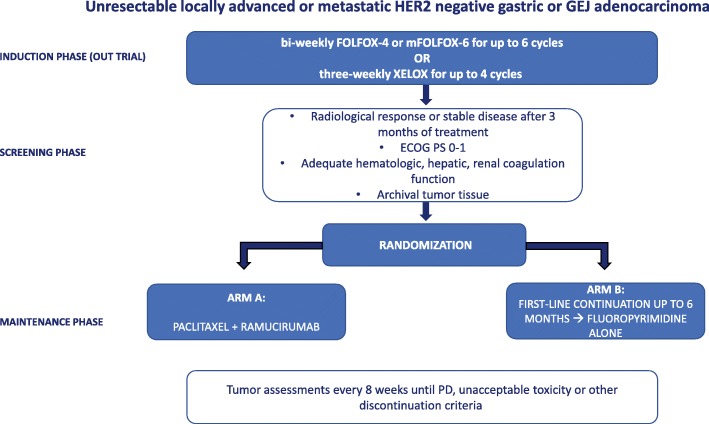
Study design

The ARMANI trial is an open label, multicenter, phase III randomized study. This is a superiority trial evaluating ramucirumab plus paclitaxel, given as switch maintenance (arm A), versus continuation of first-line chemotherapy (arm B), given as per standard clinical practice, in subjects with unresectable locally advanced or metastatic HER-2 negative gastric or GEJ cancer, without disease progression following 3 months of first-line doublet chemotherapy with fluoropyrimidine (either 5-FU or capecitabine) and oxaliplatin. Patients will be enrolled by their treating investigators and assigned to a treatment arm by 1:1 central randomization. Before randomization the patients will be stratified by center; prior gastrectomy; peritoneal carcinomatosis; site of origin. The planned population of 280 patients will be randomized at 32 study centers in Italy (see Table 1). Investigator meetings and monthly accrual updates will be held to ensure adequate enrollment. The study schema is depicted in Fig. 1.

**Table 1:** Participating Centers

Principal Investigator	Site Name
Maria Di Bartolomeo	Istituto Tumori, Milano
Giovanni Luca Frassineti	IRST, Meldola (FO)
Stefano Tamberi	IRST, Ravenna
Davide Tassinari	U.O. Ospedale degli Infermi, Rimini
Giorgio Scagliotti	AOU San Luigi Gonzaga, Orbassano (TO)
Sara Lonardi	IRCCS Istituto Oncologico Veneto, Padova
Andrea Bonetti	Ospedale Mater Salutis, Legnago (VR)
Giovanni Gerardo Cardellino	AOU, Udine
Rossana Berardi	Ospedali Riuniti, Ancona
Alberto Zaniboni	Fondazione Poliambulanza, Brescia
Mario Scartozzi	AOU Cagliari, Cagliari
Lorenza Rimassa	Humanitas Cancer Center, Rozzano (MI)
Alberto GianLuigi Luporini	IRCCS Policlinico San Donato, Milano
Giampaolo Tortora	Policlinico G.B. Rossi, Verona
Alessandro Bertolini	Azienda Ospedaliera Sondrio
Gabriele Luppi	AOU, Modena
Francesco Giuliani	Istituto Oncologico, Bari
Samantha Di Donato	H Nuovo, Prato
Evaristo Maiello	Ospedale Casa Sollievo della Sofferenza, San Giovanni Rotondo (FO)
Francesco Di Costanzo	AOU Careggi, Firenze
Graziella Pinotti	H di Circolo, Varese
Raffaella Longarini	H San Gerardo, Monza
Saverio Cinieri	H Perrino, Brindisi
Gianluca Tomasello	H Cremona, Cremona
Hector Soto Parra	P.O.G. Rodolico, Catania
Lorenzo Fornaro	AOU Pisa
Sergio Bracarda	AUSL Arezzo, Arezzo
Vincenzo Catalano	H Riuniti Marche Nord, Pesaro
Libero Ciuffreda	H Molinette, Torino
Claudio Verusio	H Saronno, Saronno (VA)
Michele Basso	Policlinico A. Gemelli, Roma
Ferdinando De Vita	AOU L. Vanvitelli, Napoli

### Study endpoints

The primary endpoint is PFS, defined as the time from randomization to the first documentation of objective disease progression or death due to any cause, whichever occurs first. Descriptive analysis of PFS will be carried out by plotting Kaplan−Meier survival curves, and median survival will be estimated. As typically done for this type of studies, superiority of the experimental treatment arm versus the control one will be established if the confidence interval upper margin of PFS difference is inferior to 1. An additional analysis will be conducted by mean of the Cox proportional hazard regression model, incorporating information on recognized prognostic factors so as to obtained an adjusted estimate of experimental treatment effect. Similar analyses will be conducted on OS.

All efficacy analyses will be primarily based on the intention-to-treat (ITT) population, and further verified on the per-protocol population.

Secondary endpoints are:

- OS, defined as the time from the first day of treatment to the date of death due to any cause. For patients still alive at the time of analysis, the OS time will be censored on the last date the patients were known to be alive.
- Time-to-treatment failure (TTF), defined as the time from the first day of treatment to the first occurrence of progressive disease and/or withdrawal due to adverse events, insufficient therapeutic response, death, lost to follow-up, refusing treatment/being unwilling to cooperate/withdrawing consent.
- Overall response rate (ORR), defined as the percentage of patients achieving complete and partial responses according to RECIST 1.1 criteria. Best overall response is the best response recorded from the start of treatment until disease progression. Complete and partial responses have to be confirmed by two evaluations of the disease.
- Duration of response (DOR), defined as the time from the initial occurrence of documented CR or PR (whichever occurs first) until documented disease progression as determined by RECIST 1.1.
- The percentage of patients that will receive a second line therapy according to arm treatment.
- Safety, assessed through summaries of adverse events graded according to NCI CTCAE 4.03

### Exploratory endpoints

As exploratory endpoints, potential biomarkers and their correlation with outcome measures will be investigated as follows: change in plasma biomarkers, such as circulating cytokines, and their correlation with outcomes (PFS, ORR, and OS); pharmacogenetic studies to find a potential correlation between single nucleotide polymorphisms and ramucirumab-related toxicity and efficacy, chemotherapy-related toxicity, drugs dose intensity and outcomes (PFS, ORR, and OS); tissue biomarkers present at baseline and investigated with next-generation sequencing (NGS). Samples will be stored at Pathology Department of Fondazione IRCCS Istituto Nazionale Tumori.

### Clinical setting

Patients affected by unresectable, locally advanced or metastatic, HER-2 negative adenocarcinoma of the stomach or GEJ can be evaluated for the study. To be considered eligible, patients must have received 3 months of first-line chemotherapy with one of the fluoropyrimidines- and oxaliplatin-based doublet combinations allowed, with radiological evidence of clinical benefit (either complete response/partial response/stable disease [CR/PR/SD] by RECIST v1.1 criteria in case of measurable disease, or absence of progressive disease in case of non-measurable disease). Patients who had received adjuvant cisplatin/oxaliplatin plus fluoropyrimidine-based doublet chemotherapy and had recurrence beyond 12 months from its completion are eligible.

Other main inclusion criteria are:

- Measurable and/or evaluable disease based on RECIST v1.1
- ECOG PS 0–1
- Adequate hematologic, hepatic, renal and coagulation function
- Available archival tumor tissue for exploratory research

Main exclusion criteria are:

- HER2 positive status, or diagnosis of squamous cell carcinoma.
- Unresolved toxicity greater than or equal to CTCAE Grade 2 attributed to any prior therapies
- Any serious illness or medical condition(s) that could be considered contraindications for any study drugs
- Treatment with any of the following, within the specified time frame, prior to study drug administration:
  *A. major* surgery within 28 days prior to randomization, or central venous access device placement within 7 days prior to randomization.
  b. Any investigational agent including VEGF or VEGFR-targeted agents within prior 4 weeks.
  c. Extended field radiation within prior 4 weeks or limited field radiation within prior 2 weeks.

### Treatment

The acceptable first-line or lead-in chemotherapy for this protocol will be a fluoropyrimidine and oxaliplatin-containing doublet (bi-weekly oxaliplatin and 5-FU [FOLFOX-4 or mFOLFOX-6 regimen]; three-weekly oxaliplatin and capecitabine [XELOX regimen]). In the induction phase, treatment must be continued for up to 4 three-weekly cycles or 6 bi-weekly cycles, or for up to a maximum of 12 weeks.

Subjects with CR/PR/SD after oxaliplatin and fluoropirimides-based regimen in case of measurable disease, or without progressive disease in case of non-measurable disease, will be randomized in 1:1 ratio between the two treatment arms:

(**Arm A)** ramucirumab 8 mg/kg on Days 1 and 15 of every 28-day cycle; paclitaxel 80 mg/m2 on Days 1, 8, and 15 of every 28-day cycle.

**(Arm B):** Continuation of the same induction regimen (FOLFOX-4 or mFOLFOX-6 for up to 6 cycles, XELOX for up to 4 cycles), followed by capecitabine or 5-FU alone.

Those on arm A will receive ramucirumab plus paclitaxel until progressive disease, unacceptable toxicity, informed consent withdrawal or patient’s death. In case of permanent discontinuation of paclitaxel due to unacceptable toxicity (particularly cumulative grade > 2 neurotoxicity), treatment with single-agent bi-weekly ramucirumab will be continued until progressive disease, unacceptable toxicity, informed consent withdrawal or patient’s death. In case of permanent discontinuation of ramucirumab due to unacceptable drug-specific toxicity, treatment with weekly paclitaxel will be continued until progressive disease, unacceptable toxicity, informed consent withdrawal or patient’s death.

Patients in arm B will receive continuation of the same regimen used as lead-in chemotherapy with the same dosage of the last cycle until progressive disease, unacceptable toxicity, informed consent withdrawal or patient’s death. Treatment will be further administered for up to 6 bi-weekly cycles in the FOLFOX schedules or for up to 4 three-weekly cycles in the XELOX schedule; then, after a maximum of 24 weeks of treatment (including both lead-in phase and post-randomization treatment), single agent fluoropyrimidines (capecitabine or 5-FU) will be continued until progressive disease, unacceptable toxicity, informed consent withdrawal or patient’s death. In case of oxaliplatin-induced severe and cumulative toxicity, prior to completion of 24-weeks intensive treatment phase, single agent fluoropyrimidine can be given as maintenance. Second-line treatment will be at Investigator’s discretion.

### Statistical design

This is a randomized, open-label, multicenter phase III trial. We plan to enroll up to 280 patients, 140 in the control group and 140 in the study group, over a two-year period. The follow up period is estimated to be of 1 year. The sample size is calculated on the basis of a superiority hypothesis of median PFS with ramucirumab plus a paclitaxel as compared to continuation of first-line CT following the randomization after the induction phase of 3 months. Taking into account a median PFS of 7 months observed in the REAL-2 trial, an overall sample size of 280 subjects (140 in the control group and 140 in the study group) achieves 90% power to detect a probability of increase of median PFS after the induction period from 4 months in the control group to 6 months in the experimental group, with a significance level of 0.05 (2-sided). Equal 1:1 allocation ratio by central randomization in the two trial arms is planned, and the patient accrual pattern over time is foreseen to be uniform. **Intention-to-treat Population:** All patients that are included in the trial by signing the informed consent and assigned a study patient number (randomized patients). **Per Protocol Population:** Patients will be excluded from the per-protocol analysis if: 1)they did not receive a minimum of 2 cycles of treatment before undergoing the first radiological reassessment, or 2) there were severe violations of protocol inclusion or exclusion criteria (for example: absence of written informed consent, HER-2 positive GC, progressive disease after the last dose of the lead-in chemotherapy).

Demographic and baseline characteristics such as age, sex, race, and baseline disease characteristics will be summarized by treatment arm for the ITT population. Descriptive baseline summaries of continuous data will present mean, standard deviation, median, minimum, and maximum. Descriptive summaries of discrete data will present the category counts as frequencies and percentages.

Descriptive analysis of PFS will be carried out by plotting Kaplan−Meier survival curves, and median survival will be estimated. As typically done for studies of this type, superiority of experimental treatment toward control will be established in case of the confidence interval upper margin of PFS difference will be inferior to 1. An additional analysis will be conducted by mean of the Cox proportional hazard regression model, incorporating information on recognized prognostic factors so as to obtained an adjusted estimate of experimental treatment effect. Similar analyses will be conducted on OS.

All efficacy analyses will be primarily based on the ITT population, and further verified on the per-protocol population.

The analysis of patients’ reported outcome (PRO- assessed using the EORTC QLQ-C30, the EORTC QLQ-OG25 and the EuroQol EQ-5D questionnaires) will be performed according to the EORTC Scoring and Reference Values Manual. All scores and subscales will be assessed through descriptive summary statistics.

For PFS, patients without a date of disease progression will be analyzed as censored observations on the date of last tumor assessment. If no post-baseline tumor assessment is available, PFS will be censored at day 1. For OS, patients who are not reported as being died will be analyzed as censored observations on the date when they were last known to be alive. If no post-baseline data are available, OS will be censored at day 1. Patients not receiving at least one dose of study drug will be excluded from the analysis of safety. Tables of adverse event incidence and individual incidence will be produced according to the primary system-organ class (SOC) and within the category defined in the CTCAE v4.03. The summaries will be overall (severity grades 1–5) and for grade ≥ 3 events. Multiple occurrences of the same event will be counted once at the maximum severity. A complementary analysis of adverse events by severity of event and by relationship to trial treatment will also be performed. The actions taken in terms of treatment discontinuation will be reported. A standard safety analysis with tables and shift tables for laboratory data will be provided.

The Trial Office of Fondazione IRCCS Istituto Nazionale dei Tumori will develop electronic case report form (eCRF) specific for this study. The Sponsor Fondazione IRCCS Istituto Nazionale dei Tumori will be responsible for data management of this study, including quality checking of the data.

## Discussion

The optimal duration of systemic first-line chemotherapy for metastatic gastric cancer is unknown. In many trials, chemotherapy was given until progression or limiting toxicity, whereas in other trials treatment was stopped at a pre-defined time. Given the increasing toxicity rate with a prolonged administration of systemic chemotherapy, patients’ quality of life could be negatively affected. Additionally, regardless the strategy of treatment, the PFS does not seem to be affected.

Given the positive results of both randomized trials and reports from clinical experiences [10, 11, 18], ramucirumab either alone or in combination with paclitaxel has proved to be a safe and active option for second line treatment in gastric cancer. Unfortunately, RAINFALL study failed to prove a clinically relevant benefit of a ramucirumab-based regimen in the first-line setting, as compared to chemotherapy doublets [19]. Nevertheless, the early administration of an active, non-cross resistant treatment after the first-line therapy, before disease progression occurs, might prolong the benefit of first-line treatment and could delay clinical deterioration [20–22]. Small phase 2 trials investigated the feasibility of sequential therapy in AGC [20–22], and they showed the potential of sequential therapy in order to prolong therapeutic benefit of first-line, but with the price of cumulative toxicities. Our hypothesis is that the early administration of a safe second-line regimen such as ramucirumab plus paclitaxel may prolong progression-free survival and consequently allow patients to experience a better quality of life.

PFS was chosen as primary endpoint instead of OS because the latter could be influenced by the best second line treatment available after trial for both arms and by the percentage of patients that are in adequate clinical condition for a second line therapy.

Many other maintenance studies, both for metastatic colorectal and lung cancers, have, in fact, PFS as primary endpoint, as it reflects the direct effect of the maintenance therapy on delaying the disease progression and it is not influenced by post progression treatments.

This topic is remarkably relevant in gastric cancer patients, in which only 40% is usually eligible for second-line treatment, independently of the first-line therapy, due to the rapid deterioration of health status after the first disease progression. The early administration of an active second-line treatment could overcome this problem and it may rescue all those subjects that become ineligible for such therapy.

Additionally, as exploratory endpoints, we will investigate several potential efficacy and toxicity biomarkers both in blood and tissue samples.

Therefore, the ARMANI study will help us at defining whether switchmaintenance with ramucirumab plus paclitaxel is a better strategy then continuation of first-line chemotherapy for HER-2 negative AGC who have not progressed after first-line platinum/fluoropyrimidine regimen.

## Abbreviation

5-FU: 5-fluoruracil
AEs: adverse events
AGC: advanced gastric cancer
AIOM: Associazione Italiana Oncologia Medica
ASCO: American Society of Clinical Oncology
BSC: best supportive care
CI: Confidence Interval
CR: Complete Response
CT: chemotherapy
DOR: duration of response
ECF: epirubicin + cisplatin + 5-FU
ECOG PS: Eastern Cooperative Oncology Group – performance status
eCRFs: electronic Case Report Forms
ECX: epirubicin + cisplatin + capecitabine
EMA: European Medicines Agency
EOF: epirubicin + oxaliplatin + 5-fluorouracil (5-FU)
EOX: epirubicin + oxaliplatin + capecitabine
ESMO: European Society for Medical Oncology
FDA: Food and Drug Administration
FOLFIRI: Folinic-acid, 5-fluorouracil, irinotecan
FOLFOX: Folinic-acid, 5-fluorouracil, Oxaliplatin
GEJ: gastroesophageal junction cancers
HR: Hazard Ratio
NCI CTCAE: National Cancer Institute Common Terminology Criteria for Adverse Events
NGS: Next-Generation Sequencing
ORR: Overall Response Rate
OS: Overall Survival
PFS: Progression Free Survival
PR: partial response
PRO: patients reported outcomes
RECIST: Response Evaluation Criteria in Solid Tumors
SD: stable disease
TTF: time to treatment failure
VEGFR2: Vascular Endothelial Growth Factor Receptor 2
XELOX: Capecitabine, Oxaliplatin
